# Supplementary material for: A New Class of Cell Wall-Recycling l,d-Carboxypeptidase Determines β-Lactam Susceptibility and Morphogenesis in Acinetobacter baumannii
Source: mBio. 2021 Dec 7;12(6):e02786-21. doi: 10.1128/mBio.02786-21 (PMC8649774; doi:10.1128/mBio.02786-21)
Supplement: FIG S1 [file mbio.02786-21-sf001.pdf]

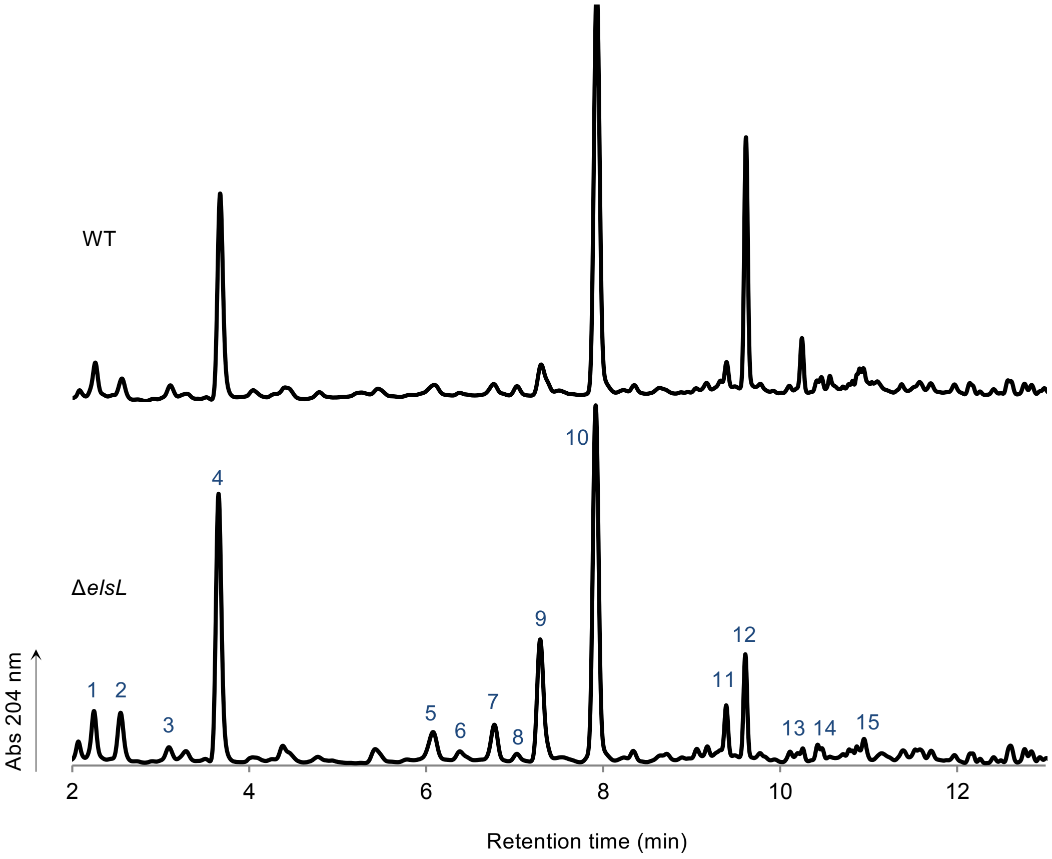

#	Label	Description	Expected m/z [M+H]+	Expected m/z [M+H]+2	Expected m/z [M+H]+3	Observed m/z	Difference
1	M3	Monomer disaccharide tripeptide	**871.3784**	436.1931	291.1314	871.3450	0.0334
2	$M4^{Gly}$	Monomer disaccharide tetrapeptide with Gly at fourth position	**928.3999**	464.7039	310.1385	928.3572	0.0427
3	$M5^{Gly}$	Monomer disaccharide pentapeptide with Gly at fifth position	**999.4370**	500.2224	333.8176	999.3960	0.041
4	M4	Monomer disaccharide tetrapeptide	**942.4155**	471.7117	314.8104	942.3746	0.0409
5	$D34^{Gly}$	Dimer disaccharide tri-tetrapeptide with Gly at fourth position	1780.7599	**890.8839**	594.2585	890.8522	0.0317
6	D33	Dimer disaccharide tri-tripeptide	1723.7384	**862.3731**	575.2514	862.3391	0.0340
7	$D44^{Gly}$	Dimer disaccharide tetra-tetrapeptide with Gly at fourth position	1851.7970	**926.4024**	617.9376	926.3626	0.0398
8	D43	Dimer disaccharide tetra-tripeptide	1794.7755	**897.8917**	598.9304	897.8583	0.0334
9	D34	Dimer disaccharide tri-tetrapeptide	1794.7755	**897.8917**	598.9304	897.8522	0.0395
10	D44	Dimer disaccharide tetra-tetrapeptide	1865.8126	**933.4102**	622.6094	933.3690	0.0412
11	T434/T344	Trimer disaccharide tetra-tri-tetrapeptide/ tri-tetra-tetrapeptide	2718.1727	1359.5903	**906.7295**	906.6955	0.0340
12	T444	Trimer disaccharide tetra-tetra-tetrapeptide	2789.2089	1395.1088	**930.4085**	930.3705	0.0380
13	Tt4444	Tetramer disaccharide tetra-tetra-tetra-tetrapeptide	3712.6069	1856.8074	**1238.2075**	1238.1420	0.0655
14	D44N	Anhydrous dimer disaccharide tetra-tetrapeptide	1845.7864	**923.3971**	615.9340	923.3565	0.0406
15	T444N	Anhydrous trimer disaccharide tetra-tetra-tetrapeptide	2769.1836	1385.0957	**923.7331**	923.6984	0.0347
