# Supplementary figures and images for: A New Class of Cell Wall-Recycling l,d-Carboxypeptidase Determines β-Lactam Susceptibility and Morphogenesis in Acinetobacter baumannii
Source: mBio. 2021 Dec 7;12(6):e02786-21. doi: 10.1128/mBio.02786-21 (PMC8649774; doi:10.1128/mBio.02786-21)

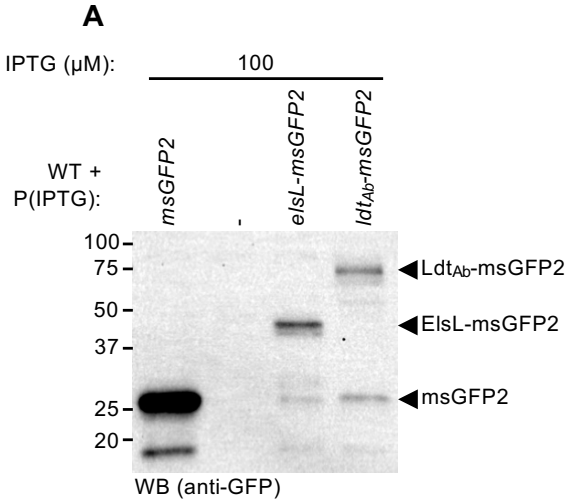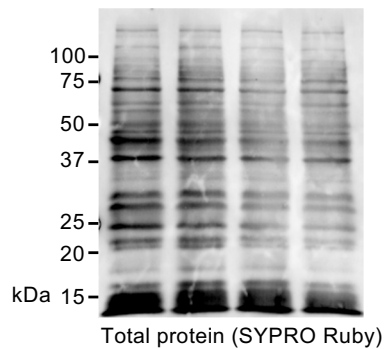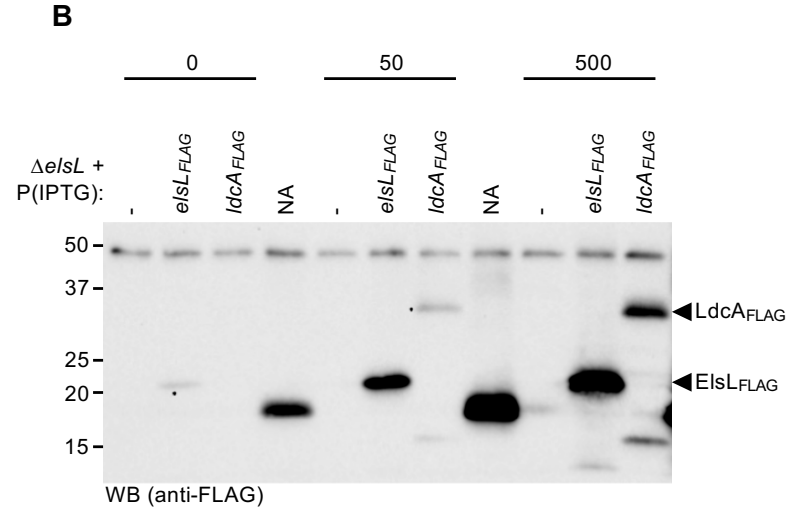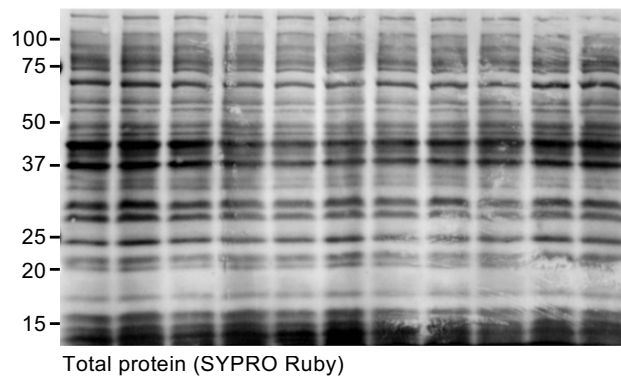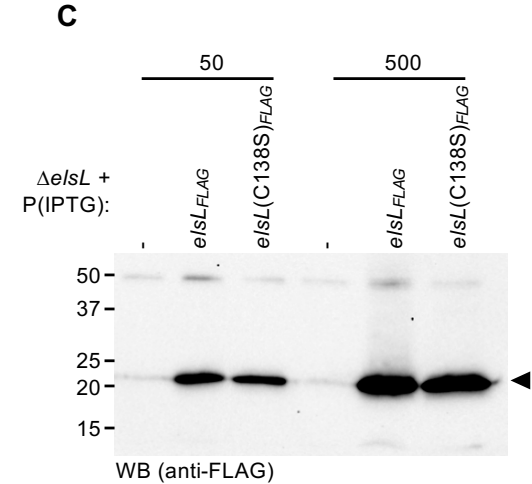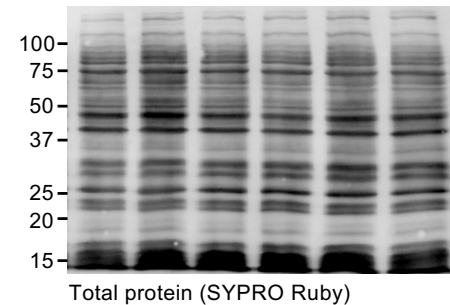

Supplement: FIG S2 [file mbio.02786-21-sf002.pdf]

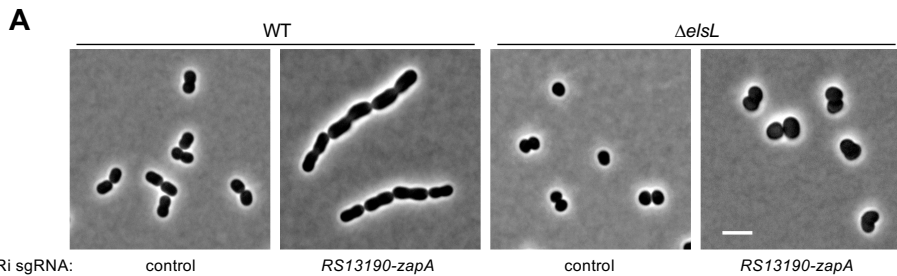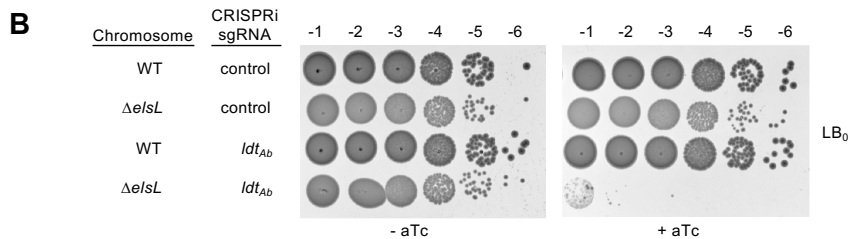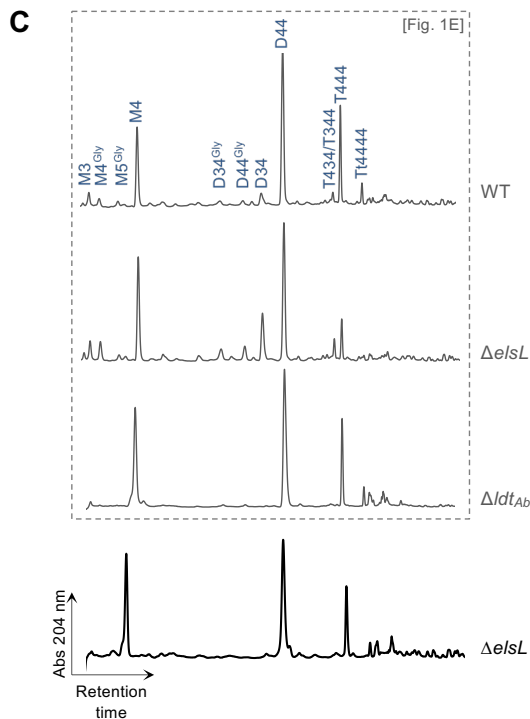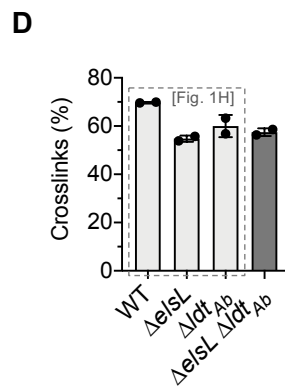

Supplement: FIG S3 [file mbio.02786-21-sf003.pdf]

**A**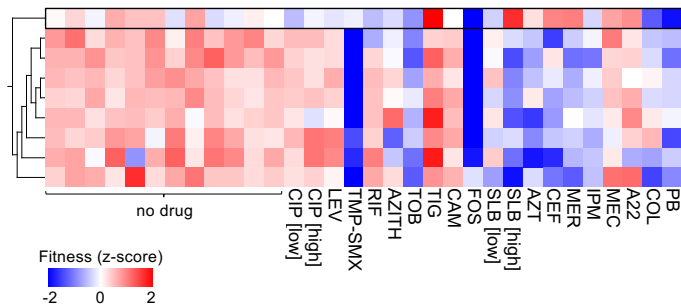**B**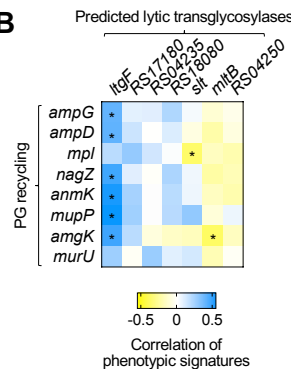**C**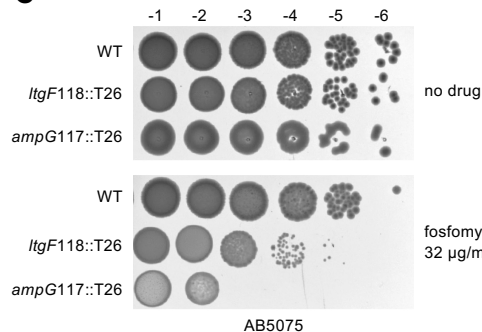**D**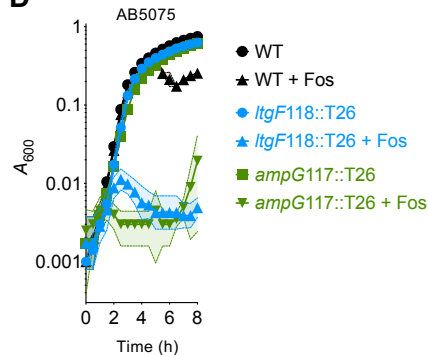**E**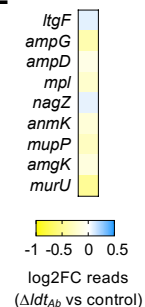

Supplement: FIG S4 [file mbio.02786-21-sf004.pdf]

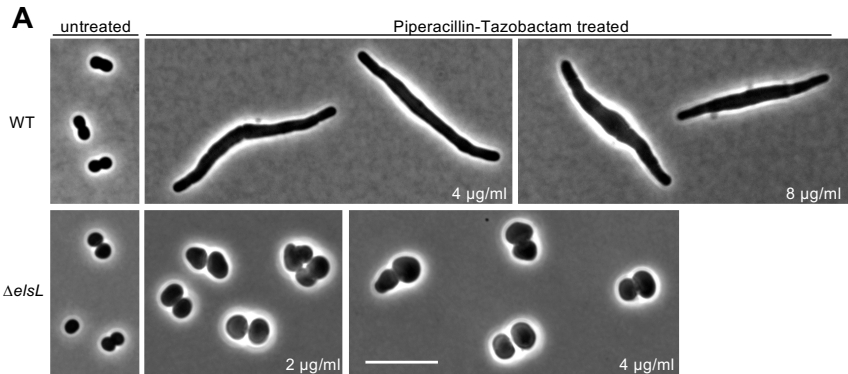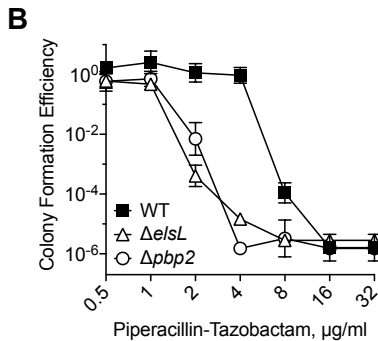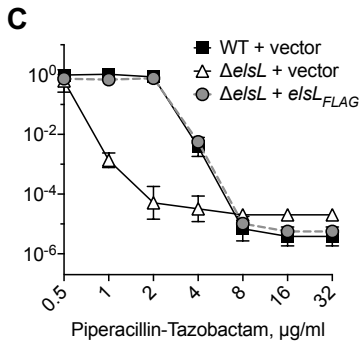

Supplement: FIG S5 [file mbio.02786-21-sf005.pdf]
