# Supplementary material for: A New Class of Cell Wall-Recycling l,d-Carboxypeptidase Determines β-Lactam Susceptibility and Morphogenesis in Acinetobacter baumannii
Source: mBio. 2021 Dec 7;12(6):e02786-21. doi: 10.1128/mBio.02786-21 (PMC8649774; doi:10.1128/mBio.02786-21)
Supplement: TABLE S1 [file mbio.02786-21-st001.pdf]

Table S1. Quantification of genetic interactions with ∆*elsL*.

	sgRNA: control	*ldt_{Ab}*	*RS13190*	*blhA*	*mlaF*
Growth phenotype of single CRISPRi strain (A)[1]	1.00 (n = 20)	1.00 (n = 12)	0.8 (n = 10)	1.03 (n = 9)	0.83 (n = 7)
Growth phenotype of single ∆*elsL* strain (B)[2]	0.87 (n = 16)	–	–	–	–
Expected growth phenotype of double lesion strain (A*B)	–	0.87	0.69	0.90	0.73
Observed growth phenotype of double lesion strain[3]	–	0.28* (n = 8)	n.d.	0.56* (n = 5)	0.47* (n = 5)

[1]Mean colony diameter, relative to WT control, of single CRISPRi lesion (sgRNA in WT background)
[2]Mean colony diameter, relative to WT control, of single ∆*elsL* lesion (control sgRNA in ∆*elsL* background)
[3]Mean colony diameter, relative to WT control, of double lesion (sgRNA in ∆*elsL* background)
n, number of measured colonies
–, not applicable
n.d., colonies not detected
*, $p < 0.001$ in one sample t test using the expected growth phenotype as reference value.
