# Supplementary material for: A New Class of Cell Wall-Recycling l,d-Carboxypeptidase Determines β-Lactam Susceptibility and Morphogenesis in Acinetobacter baumannii
Source: mBio. 2021 Dec 7;12(6):e02786-21. doi: 10.1128/mBio.02786-21 (PMC8649774; doi:10.1128/mBio.02786-21)
Supplement: TABLE S2 [file mbio.02786-21-st002.pdf]

Table S2. Strains, plasmids, and primers used in this study

Strains

**Strain**	**Genotype or description**	**Reference**
A. baumannii		
ATCC 17978	cerebrospinal fluid isolate	(1)
EGA738	ATCC 17978 Δ*elsL* (ACX60_RS03475)	(2)
EGA739	ATCC 17978 Δldt_{Ab} (ACX60_RS05685)	(2)
EGA740	ATCC 17978 Δ*elsL* Δldt_{Ab}	This study
YDA350	ATCC 17978 with pYDE153 (vector)	This study
YDA475	EGA738 (Δ*elsL*) with pEGE305 (vector)	This study
YDA515	EGA738 (Δ*elsL*) with pYDE350 (P(IPTG)-*elsL*-3xFLAG)	This study
YDA516	EGA738 (Δ*elsL*) with pYDE351 (P(IPTG)-*ldcA*-3xFLAG)	This study
YDA519	EGA739 (Δldt_{Ab}) with pEGE305 (vector)	This study
YDA518	EGA739 (Δldt_{Ab}) with pYDE240 (P(IPTG)-ldt_{Ab})	This study
YDA004	ATCC 17978 *attTn7*::*tet*R-*tet*P-*dcas9*-*rrnB*T1-T7Te Gm^r	(3)
YDA007	YDA004 with pYDE007 (non-targeting control)	(3)
YDA232	YDA004 with pYDE044 ($sgRNA_{ldtAb}$)	This study
YDA549	YDA004 with pYDE381 ($sgRNA_{blhA}$)	This study
YDA535	YDA004 with pYDE376 ($sgRNA_{RS13190}$)	This study
YDA533	YDA004 with pYDE374 ($sgRNA_{mlaF}$)	This study
YDA186	ATCC 17978 Δ*elsL* *attTn7*::*tet*R-*tet*P-*dcas9*-*rrnB*T1-T7Te Gm^r	This study
YDA281	YDA186 with pYDE007 (non-targeting control)	This study
YDA284	YDA186 with pYDE044 ($sgRNA_{ldtAb}$)	This study
YDA550	YDA186 with pYDE381 ($sgRNA_{blhA}$)	This study
YDA539	YDA186 with pYDE376 ($sgRNA_{RS13190}$)	This study
YDA537	YDA186 with pYDE374 ($sgRNA_{mlaF}$)	This study
YDA315	EGA740 (Δ*elsL* Δldt_{Ab}) *ampG*(W99*)	This study
YDA331	EGA740 (Δ*elsL* Δldt_{Ab}) *mpl*(A335T)	This study
YDA380	EGA740 (Δ*elsL* Δldt_{Ab}) *ltgF::IS*	This study
EGA516	ATCC 17978 Δ*ampG*	(4)
EGA692	ATCC 17978 Δ*pbp2*	(4)
YDA414	ATCC 17978 Δ*elsL* Δldt_{Ab} Δ*ampG*	This study
YDA411	ATCC 17978 Δ*elsL* Δ*ampG*	This study
AB5075-UW	bone isolate/osteomyelitis	(5)
AB07436	AB5075-UW *ltgF*118::T26	(5)
AB03078	AB5075-UW *ampG*117::T26	(5)
E. coli		
DH5α	*supE44* Δ*lacU169* (ϕ80lacZΔM15) *hsdR17 recA1 endA1 gyrA96 thi*-1 *relA1*	(6)
DH5λpir	DH5α (λpir) *tet*::Mu *recA*	(7)
XL1-blue	*recA1 endA1 gyrA96 thi-1 hsdR17 supE44 relA1 lac* [F′ *proAB* $lacI^q$ZΔM15 Tn10 Tc^r)]	Stratagene

Plasmids

Plasmid	Description	Reference
pUC18	*oriColE1* MCS Cb^r	(8)
pSR47S	Conditionally replicating allele exchange plasmid, Km^r (*oriTRP4 oriR6K sacB* Km^r)	(9)
pJB4648	Conditionally replicating allele exchange plasmid, Gm^r (*oriTRP4 oriR6K sacB* Gm^r)	(9)
pEGE305	P(IPTG) shuttle vector (*ori-pBR322 ori-pWH1277 bla*::*lacI*^{q}-T5*lac*P, Tc^r)	(4)
pYDE153	P(IPTG) shuttle vector (*ori-pBR322 ori-pWH1277 bla*::*lacI*^{q}-T5*lac*P-MCS, Tc^r)	(2)
pDL1100	*Himar1 mariner* (Km^r) delivery plasmid, C9 transposase, *ori pSC101* Cb^r	(2)
pYDE009	*dcas*9 miniTn7 delivery plasmid (*oriColE1* miniTn7::*tetR*-tetP-*dcas9*-*rrnB*T1-T7Te, Gm^r, Cb^r)	(3)
pYDE007	sgRNA delivery plasmid non-targeting control guide (*ori-pBR322 ori-pWH1277* P_{J23119}-sgRNA$_{mrfp}$-terminator, Cb^r)	(3)
pYDE044	pYDE007 derivative with sgRNA$_{ldtAb}$	This study
pYDE381	pYDE007 derivative with sgRNA$_{blhA}$	This study
pYDE376	pYDE007 derivative with sgRNA$_{RS13190}$	This study
pYDE374	pYDE007 derivative with sgRNA$_{mlaF}$	This study
pEGE268	pSR47S::Δ*elsL* allele exchange construct	(2)
pEGE271	pSR47S::Δ*ldt*$_{Ab}$ allele exchange construct	(2)
pYDE388	pJB4648::Δ*ltgF* allele exchange construct	This study
pYDE231	pUC18::*ldt*$_{Ab}$	This study
pYDE240	pEGE305::*ldt*$_{Ab}$ (P(IPTG)-*ldt*$_{Ab}$)	This study
pYDE350	pEGE305::*elsL-3xFLAG* (P(IPTG)-*elsL-3xFLAG*)	This study
pYDE351	pEGE305::*ldcA-3xFLAG* (P(IPTG-*ldcA-3xFLAG*)	This study
pYDE342	pUC18::*elsL* (XbaI hexameric sequence in-frame)	This study
pYDE063	pUC18::*ldt*$_{Ab}$ (XbaI hexameric sequence in-frame)	This study
pYDE343	pUC18::*ldcA* (XbaI hexameric sequence in-frame)	This study
pYDE346	pUC18::*elsL-3xFLAG*	This study
pYDE347	pUC18::*ldcA-3xFLAG*	This study
pYDE386	pUC18::*elsL-msGFP2*	This study
pYDE387	pUC18::*ldt*$_{Ab}$*-msGFP2*	This study
pYDE389	pEGE305::*elsL-msGFP2* (P(IPTG)-*elsL-msGFP2*)	This study
pYDE390	pEGE305::*ldt*$_{Ab}$*-msGFP2* (P(IPTG)-*ldt*$_{Ab}$*-msGFP2*)	This study
pYDE281	pUC18::*elsL-6xHis*	This study
pYDE324	pUC18::*ldcA-6xHis*	This study
pYDE290	pET28b::*elsL-6xHis*	This study
pYDE328	pET28b::*ldcA-6xHis*	This study

Oligonucleotide primers and gene fragment

Primer or fragment name	Sequence (5' – 3'; restriction site underlined if present)	RE site(s)
Complementation and localization experiments		
Bam-elsL-F	GATATCGGATCCAGTTGAGTTGATATAAGAGAAGAAATTCG	BamHI
Xba-elsL-R	GATCATTCTAGAGGTTAAAGATTGTTCAGACAAATAAAC	XbaI
Bam-ldcA-F	GATATCGGATCCAGTTGAGTTGATATAAGAGAAGAAATTCGATGTCTCTGTTTCACTTAATTGC	BamHI
Xba-ldcA-R	GATCATTCTAGACATTTTAAGAACAGGATGACCGC	XbaI
ldtAb-BamHI-F	GATATCGGATCCGCTTATATGAAAATTCTTAAGGTTG	BamHI
ldtAb-XbaI-R	GATCATTCTAGAATTCTAAGAATTTAACAGTTACGCC	XbaI
Ldt-OE-F	TGCATAGAATTCCAAAAGTCTACTGCAAGAAGAAACG	EcoRI
Ldt-OE-R	GCTGAACTGCAGGAAACAGAAGCTAAAATGCAAACC	PstI
elsL-C138S-F	TAGATTCAATTGTGAGCGGATAACAATTTC	
elsL-C138S-R	GATCATTCGAATAGACCCATGTGACATTGGAAC	BstBI
msGFP2_F	GTCAGAATTCTTTAAGAAGGAGATATACATATGGATTCTACTGAATCTTTATTCAC	EcoRI
msGFP2_R	AACTGCAGGTCTGGACATTTAAG	PstI
Gene deletion		
D-RS13100-up-F	TTCGTGGATCCCAGTAATACCGTGGACTTGAACAG	BamHI
D-RS13100-up-R	GGGAGGGTACCTGGTTTATACATAAAAACTTCCAAACAAC	KpnI
D-RS13100-dw-F	CTGAGGGTACCGATATCATTAAAGTGCCTAATTTATAG	KpnI
D-RS13100-dw-R	CTTGCGCGGCCGCCTTTGTAGTCTTTGATATTGTCC	NotI
Overexpression and purification of elsL* and *ldcA		
elsL-NcoI-FOR	TGCATACCATGGCTCAAAATAGTTGAGTTGATATAAGAG	NcoI
elsL-his-EcoRI-REV	GATCATGAATTCTTAGTGGTGGTGGTGGTGGTGGGTTAAAGATTGTTCAGACAAATAAAC	EcoRI
ldcA-NcoI-FOR	TGCATACCATGGCATGATATACATTTCGTTTCTGTTGTC	NcoI
ldcA-his-EcoRI-REV	GATCATGAATTCTTAGTGGTGGTGGTGGTGGTGCATTTTAAGAACAGGATGACCGCT	EcoRI
CRISPRi		
SGR29-F	TTACACTAGTAGCAGATACTTTATTAGGGTTCAGGTTTTAGAGCTAGAAATAGCAAG	SpeI
SGR45-F	TTACACTAGTTTGATAAGCATGACGGACAGCTTCGTTTTAGAGCTAGAAATAGCAAG	SpeI
SGR60-F	TTACACTAGTCGCTGTAATTGTTCTAACATAACGGTTTTAGAGCTAGAAATAGCAAG	SpeI
SGR58-F	TTACACTAGTAGAGAGGAGTTTTATTATTCATAAGTTTTAGAGCTAGAAATAGCAAG	SpeI
SGR-R (BglII) (ref. 3)	AAGTGGGCCCAGATCTAAGCTTCAAAAAAAGCACCGAC	ApaI,<br>BglII
Gene fragment		
msGFP2<br>(for C-terminal fusion, codon optimized)	AGTTCGTCTAGAGGCGGCGGCGGCGGCATGGATTCTACTGAATCTTTATTCACTGGTGTTGT<br>TCCAATTTTAGTTGAATTAGATGGTGATGTTAACGGTCACAAATTCTCTGTTCGTGGTGAAGG<br>TGAAGGTGATGCTACTAACGGTAAATTAACTTTAAAATTCATTTGTACTACTGGTAAATTACCA<br>GTTCCATGGCCAACTTTAGTTACTACTTTAACTTATGGTGTTCAATGTTTCTCTCGTTATCCAG<br>ATCACATGAAACAACACGATTTCTTCAAATCTGCTATGCCAGAAGGTTATGTTCAAGAACGTA<br>CTATTACTTTCAAAGATGATGGTACTTATAAAACTCGTGCTGAAGTTAAATTCGAAGGTGATA<br>CTTTAGTTAACCGTATTGAATTAAAAGGTATTGATTTCAAAGAAGATGGTAACATTTTAGGTCA<br>CAAATTAGAATATAACTATAACTCTCACAACGTTTATATTACTGCTGATAAACAAAAAAACGGT<br>ATTAAAGCTAACTTCAAAATTCGTCACAACGTTGAAGATGGTTCTGTTCAATTAGCTGATCACT<br>ATCAACAAAACACTCCAATTGGTGATGGTCCAGTTTTATTACCAGATAACCACTATTTATCTAC<br>TCAATCTAAATTATCTAAAGATCCAAACGAAAAACGTGATCACATGGTTTTATTAGAGTTTGTT<br>ACTGCTGCTGGTATTACTGGTGGTTCTGGTGGTTCTTAAATGTCCAGACCTGCAGTTCTG	XbaI,<br>PstI

Supplemental References

1. Bouvet P, Grimont P. 1986. Taxonomy of the Genus *Acinetobacter* with the Recognition of *Acinetobacter baumannii* sp. nov. *Acinetobacter haemolyticus* sp. nov. *Acinetobacter johnsonii* sp. nov. and *Acinetobacter junii* sp. nov. and Emended Descriptions of *Acinetobacter calcoaceticus* and *Acinetobacter lwofii*. International Journal of Systematic Bacteriology 36:228-240.
2. Geisinger E, Mortman NJ, Dai Y, Cokol M, Syal S, Farinha A, Fisher DG, Tang AY, Lazinski DW, Wood S, Anthony J, van Opijnen T, Isberg RR. 2020. Antibiotic susceptibility signatures identify potential antimicrobial targets in the *Acinetobacter baumannii* cell envelope. Nature Communications 11:4522.
3. Bai J, Dai Y, Farinha A, Tang AY, Syal S, Vargas-Cuebas G, van Opijnen T, Isberg RR, Geisinger E. 2021. Essential gene analysis in *Acinetobacter baumannii* by high-density transposon mutagenesis and CRISPR interference. J Bacteriol 203.
4. Geisinger E, Mortman NJ, Vargas-Cuebas G, Tai AK, Isberg RR. 2018. A global regulatory system links virulence and antibiotic resistance to envelope homeostasis in *Acinetobacter baumannii*. PLoS Pathog 14:e1007030.
5. Gallagher LA, Ramage E, Weiss EJ, Radey M, Hayden HS, Held KG, Huse HK, Zurawski DV, Brittnacher MJ, Manoil C. 2015. Resources for Genetic and Genomic Analysis of Emerging Pathogen *Acinetobacter baumannii*. J Bacteriol 197:2027-35.
6. Hanahan D, Jessee J, Bloom FR. 1991. Plasmid transformation of *Escherichia coli* and other bacteria. Methods Enzymol 204:63-113.
7. Kolter R, Inuzuka M, Helinski DR. 1978. Trans-complementation-dependent replication of a low molecular weight origin fragment from plasmid R6K. Cell 15:1199-208.
8. Yanisch-Perron C, Vieira J, Messing J. 1985. Improved M13 phage cloning vectors and host strains: nucleotide sequences of the M13mp18 and pUC19 vectors. Gene 33:103-19.
9. Andrews HL, Vogel JP, Isberg RR. 1998. Identification of linked *Legionella pneumophila* genes essential for intracellular growth and evasion of the endocytic pathway. Infect Immun 66:950-8.
